# Clinical application of prion-like seeding in α-synucleinopathies: Early and non-invasive diagnosis and therapeutic development

**DOI:** 10.3389/fnmol.2022.975619

**Published:** 2022-10-10

**Authors:** Jiaqi Li, Haiyang Luo, Honglin Zheng, Suying Duan, Taiqi Zhao, Yanpeng Yuan, Yutao Liu, Xiaoyun Zhang, Yangyang Wang, Jing Yang, Yuming Xu

**Affiliations:** ^1^Department of Neurology, The First Affiliated Hospital of Zhengzhou University, Zhengzhou University, Zhengzhou, Henan, China; ^2^Henan Key Laboratory of Cerebrovascular Diseases, The First Affiliated Hospital of Zhengzhou University, Zhengzhou University, Zhengzhou, Henan, China; ^3^Institute of Neuroscience, Zhengzhou University, Zhengzhou, Henan, China; ^4^The Academy of Medical Sciences of Zhengzhou University, Zhengzhou University, Zhengzhou, Henan, China

**Keywords:** α-synuclein, RT-QuIC, prion, Parkinson’s disease, multiple-system atrophy, dementia with Lewy bodies

## Abstract

The accumulation and deposition of misfolded α-synuclein (α-Syn) aggregates in the brain is the central event in the pathogenesis of α-synucleinopathies, including Parkinson’s disease, dementia with Lewy bodies, and multiple-system atrophy. Currently, the diagnosis of these diseases mainly relies on the recognition of advanced clinical manifestations. Differential diagnosis among the various α-synucleinopathies subtypes remains challenging. Misfolded α-Syn can template its native counterpart into the same misfolded one within or between cells, behaving as a prion-like seeding. Protein-misfolding cyclic amplification and real-time quaking-induced conversion are ultrasensitive protein amplification assays initially used for the detection of prion diseases. Both assays showed high sensitivity and specificity in detection of α-synucleinopathies even in the pre-clinical stage recently. Herein, we collectively reviewed the prion-like properties of α-Syn and critically assessed the detection techniques of α-Syn-seeding activity. The progress of test tissues, which tend to be less invasive, is presented, particularly nasal swab, which is now widely known owing to the global fight against coronavirus disease 2019. We highlight the clinical application of α-Syn seeding in early and non-invasive diagnosis. Moreover, some promising therapeutic perspectives and clinical trials targeting α-Syn-seeding mechanisms are presented.

## Introduction

α-Synuclein (α-Syn) is the most abundant protein found in Lewy bodies (LBs), a hallmark of α-synucleinopathies (Hansen and Li, 2012). These disorders include Parkinson’s disease (PD), dementia with LBs (DLB), and multiple-system atrophy (MSA) (Borghammer et al., 2016). Currently, the diagnosis of these diseases mainly relies on the recognition of advanced clinical symptoms. Differential diagnosis among the various α-synucleinopathies subtypes remains challenging because of overlapping symptoms. A definite diagnosis of α-synucleinopathies is essential because they have different disease mechanisms, and ultimately different therapeutic targets and prognoses. Therefore, determining methods that can provide an accurate and early diagnosis of α-synucleinopathies is crucial.

Protein misfolding and abnormal aggregation into fibrillar structures may be the key to the pathogenesis of some neurodegenerative diseases. Therefore, misfolded α-Syn into fibrillary aggregates may contribute to the development of PD. Although the pathological α-Syn present in different biological fluids, the concentrations are often well below the limits of conventional biochemical detection techniques. α-Syn can template its native counterpart into the same misfolded structure within or between cells, behaving as a prion-like seeding. In the last few years, innovative techniques, such as real-time quaking-induced conversion (RT-QuIC) and protein-misfolding cyclic amplification (PMCA), have been exploited to detect prion-seeding activity *in vitro* and make it possible to detect trace amounts of prion proteins in tissues and biological fluids. Given the prion-like properties of α-Syn, these assays have also shown high sensitivity and specificity in detecting α-synucleinopathies, even in peripheral tissues and biofluids at pre-clinical phases. Moreover, different conformational strains of α-Syn aggregates have been amplified and detected to distinguish MSA from other α-synucleinopathies by RT-QuIC, in recent studies (Shahnawaz et al., 2020).

In the present review, we critically assess the structure of α-Syn and its prion-like properties, as well as the current methods for the non-invasive neuropathological analysis of α-Syn seeding. We highlight the clinical application and significance of α-Syn seeding in early diagnosis and discrimination of α-Syn strains for the differential diagnosis between α-synucleinopathies. Moreover, possible effective therapeutic avenues and clinical trials targeting α-Syn-seeding mechanisms are presented.

## α-Syn structure and its prion properties

**FIGURE 1:**
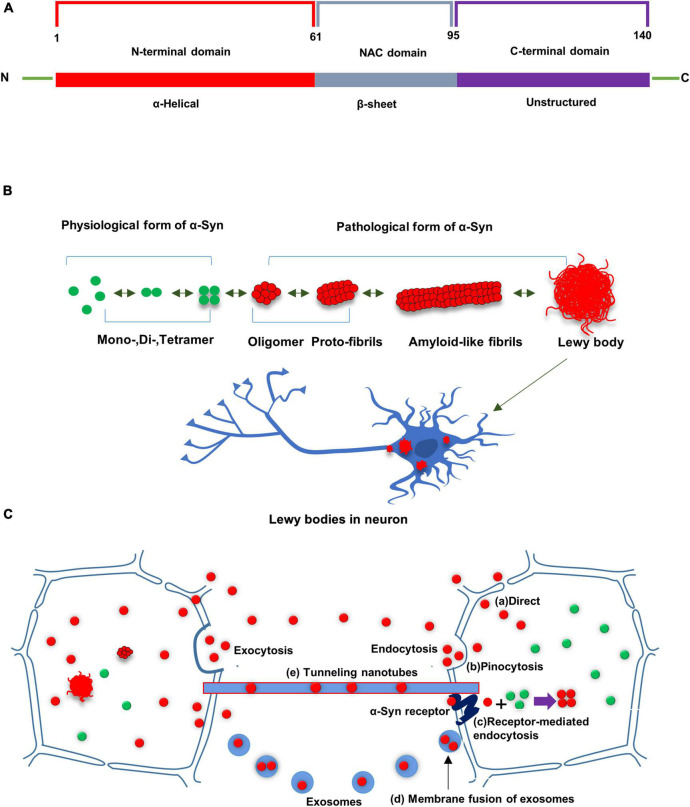
**(A)** Schematic diagram of α-Syn. α-Syn consists of three distinctive domains: N terminal domain, non-amyloid component domain (NAC), and C-terminal domain. **(B)** Native α-Syn exists in the form of monomer, dimer and tetramer in a physiological state. α-Syn changes its conception under certain conditions to form unstable oligomers and fibrils, amyloid-like fibers, and finally form insoluble protein aggregates which deposit in neurons and cause neurotoxicity. **(C)** Possible routes for cell-to-cell transfer of misfolded α-Syn: Misfolded α-Syn are secreted from donor cells to the interstitial compartment as naked protein or in vesicles (e.g., exosomes). α-Syn could be taken up by neighboring cells in culture via several routes, including direct penetration of the plasma membrane, receptor-mediated endocytosis or fusion of plasma-exosomal membranes. Tunneling nanotubes have been also shown to transport misfolded α-Syn between cells. Misfolded α-Syn from the donor cell can seed conversion of native α-Syn into aggregated misfolded α-Syn in recipient cell.

α-Syn, encoded by the *SNCA* gene, has a molecular weight of 14.5 kDa and contains 140 amino acid residues (Chen et al., 1995). The primary protein sequence consists of three distinct regions. The N-terminal domain (1–60 residues) is defined as an amphipathic α-helical structure, involved in an interaction with presynaptic lipid membranes. The NAC domain (61–95 residues), enriched in hydrophobic residues, predisposes toward pathological aggregation. The carboxyl-terminal domain (96–140 residues) does not contain a secondary structure, incorporating most post-translational modification sites (Oueslati, 2016), and the aggregated proclivity of the phosphorylation site (Figure 1A). Native α-Syn mainly exists as unfolded monomer, dimer or tetramer, all of which can be converted into partially folded conformation when exposed to pathological stimulations. It changes its conception under certain conditions such as *SNCA* mutations, inflammation, post-translational modifications, to form unstable oligomers and fibrils, amyloid-like fibers, and finally form insoluble protein aggregates which deposit in neurons and cause neurotoxicity (Figure 1B).

Although the specific formation pathway of α-Syn aggregates is still unclear, some researchers believe that oligomerization and aggregation of α-Syn are related to the binding of α-Syn to lipid membranes (Burré et al., 2018; Killinger et al., 2019). Lipid membranes promote the aggregation of α-Syn, and α-Syn oligomers lead to increased membrane permeabilization and disrupt membrane integrity (Suzuki et al., 2018). Tetramer is the physiological state of α-Syn, and α-Syn tetramer destabilization is also an early step in the aggregation of α-Syn (Nuber et al., 2018). Genetic evidence suggests that patients with duplication and triplication of the *SNCA* gene suffer from increased levels of premature aggregation of α-Syn, and more neuronal death (Farrer et al., 2004; Byers et al., 2011). In addition to genetic mutations, C-terminal truncation, including δ123-140 and δ104-140, also enhances α-Syn aggregation to some extent (Ohgita et al., 2022). In patients with neurodegenerative diseases, the oxidation-antioxidant system is out of balance, resulting in limited scavenging of reactive oxygen species (ROS) and excessive accumulation of ROS, which is also one of the factors accelerating α-Syn oligomerization (Garcia-Garcia et al., 2012). Moreover, it has been reported that exposure to various heavy metals (Pb, Mn, Fe, Mg, Zn, Al, Cu) also induces increased ROS production and directly promotes α-Syn aggregation (Santner and Uversky, 2010). α-Syn experiences diverse post-translational modifications, such as phosphorylation, ubiquitination, acetylation, nitrification, etc. (Zhang et al., 2019). This post-translational modification affects toxicity and aggregation tendency of α-Syn. Among them, phosphorylation is the most common post-translational modification, which can promote protein aggregation to form oligomers.

α-Syn possesses prion-like properties. The prion hypothesis dictates that aberrant α-Syn proteins aggregate together and induce conformational misfolding in α-Syn monomers, causing nucleation-dependent, autocatalytic amplification of α-Syn. The entire seeding process requires a sequence-specific template and a typical cross β-sheet conformation. The necessity for the presence of templates and β-sheet conformations is demonstrated by stably overexpressing α-Syn cell lines lacking the NAC region, which consists of β-sheet structures and is a hydrophobic sequence necessary for α-Syn aggregation. Addition of α-Syn pre-fibrils to these cells did not induce the formation of intracellular inclusion bodies, indicating that the seeding process requires not only a specific template but also a typical crossed β-sheet conformation. During continuous seeding, misfolded α-Syn propagates through the central nervous system (CNS) and causes extensive damage to neurons, leading to disease.

The prion-like properties of α-Syn have been supported by several studies. In 2008, α-Syn LBs were found in fetal dopaminergic neuronal grafts after transplantation into patients with PD, suggesting that the pathological α-Syn had spread from diseased neurons to the grafted neurons in the recipients’ brains (Kordower et al., 2008). Desplats et al. (2009) found that misfolded α-Syn can transfer between cultured neurons and induce the aggregation of α-Syn in recipient cells, indicating that α-Syn has prion-like properties. Since then, more and more evidence has supported the prion-like properties of α-Syn. For example, intracranial injection of α-Syn pre-fibrils prepared *in vitro* (Danzer et al., 2009; Hansen et al., 2011; Volpicelli-Daley et al., 2011; Luk et al., 2012a,b; Guo et al., 2013; Sacino et al., 2014) or brain tissue homogenates from α-Syn transgenic mice (Mougenot et al., 2012) or post-mortem PD brains (Recasens et al., 2014) could trigger aggregation formation, inflammation, neurodegeneration.

Furthermore, regarding cell-to-cell transfer of α-Syn, some studies have reported that α-Syn is secreted from donor cells into the extracellular space as naked proteins or in vesicles (e.g., exosomes) (Jang et al., 2010), and secretion of α-Syn can increase due to endoplasmic reticulum stress and membrane damage. Once entering the extracellular space, α-Syn could be taken up by neighboring cells through several pathways, comprising direct plasma membrane penetration, receptor-mediated endocytosis, or fusion of plasma-exosomal membranes (Desplats et al., 2009; Hansen et al., 2011). What’s more, cell-to-cell transfer through tunneling nanotubes, observed in prion proteins, can be a possible way for α-Syn transfer (Gousset et al., 2009) (Figure 1C).

The specific mechanism that misfolded α-Syn causes toxicity remains unclear, however, mitochondrial impairment, endoplasmic reticulum stress, increase in membrane permeability, disruption of calcium homeostasis, inhibition of SNARE complex formation, reduced dopamine release, and decreased expression of the MEF2D transcription factor have been considered (Wong and Krainc, 2017). Pathological α-Syn can also trigger the microglial inflammatory response and the production of reactive oxygen species (ROS) through a series of pathways, thereby promoting neuronal damage.

## Techniques available to detect α-Syn seeding

Oligomers are aggregation intermediates of insoluble protein aggregates. In neurodegenerative diseases oligomers and aggregates of α-Syn that are responsible for driving toxicity can be used as a template to cause conformational misfolding in α-Syn monomer, inducing template-dependent, autocatalytic amplification of α-Syn. The misfolded α-Syn can be further spread to the extracellular space and seed between the adjacent cells, indicating that they all have prion-like properties, that is, seeding properties. Phosphorylation, the most widely studied post-translational modification, is known to promote protein aggregation. Although the specific mechanism that phosphorylated α-Syn promotes the formation of oligomers, pro-fibrils, and insoluble aggregates of α-Syn remains vague, it is undeniable that phosphorylated α-Syn is closely related to α-Syn seeding activity. Therefore, various traditional and new techniques can detect α-Syn seeding activity directly and indirectly. The traditional methodologies of α-Syn-seeding assays include PK-assisted detection of α-Syn aggregate, detection of α-Syn aggregate by immunohistochemistry *in situ*, and detection of the misfolded isoforms in biofluids by special antibodies, from well-established IHC and sandwich enzyme-linked immunosorbent assays (ELISAs) to novel proximity ligation assays (PLAs). These traditional methodologies do not directly reflect the seeding activities; novel assessments directly utilizing the seeding propensity of misfolded α-Syn were explored. The most prominent assays are the PMCA and RT-QuIC, which template the conformational change in native protein and monitor the misfolded protein aggregates in real time using a fluorescent dye.

### Immunohistochemistry

Lewy bodies (LBs) and LNs, which primarily comprise of misfolded α-Syn, are major hallmarks in the brain and peripheral tissues of α-synucleinopathies. The major component of Lewy pathology is abnormally phosphorylated α-Syn. As mentioned earlier, phosphorylated α-Syn may promote the conformational alteration and pathological aggregation of α-Syn. Therefore, IHC of phosphorylated α-Syn, a technique that can detect the morphology of pathologic aggregates in skin nerves, has been also developed. Donadio et al. (2014, 2016) showed that intraneural phosphorylated α-Syn is a reliable marker of PD, and they found that phosphorylated α-Syn distinguishes PD from other parkinsonism. In addition, rapid eye movement sleep behavior disorder (RBD) is a prodromal symptom of PD. They found that 75% of RBD patients had phosphorylated α-Syn deposition, while healthy controls did not, suggesting that skin phosphorylated α-Syn may be a precursor marker of PD (Antelmi et al., 2017). However, the positive rate of phosphorylated α-Syn varied greatly for each experiment, hence, a consensus on which the most specific and sensitive location for biopsy should be drawn. Recently, Liu et al. provided an optimized phosphorylated α-Syn detection method (Liu et al., 2020). They proposed the appropriate site and the least sample required to achieve both high sensitivity and specificity, which provides a reference for the sampling site and sample size between laboratories.

### Proximity ligation assay

Proximity ligation assay (PLA) is a unique immunoassay, which can be used to detect target proteins, protein interactions and so on. In this method, the target protein is identified by a pair of probes labeled with a monoclonal or polyclonal antibody with attached DNA strands, namely PLA probe. When the two probes recognize the same protein, the distance between the two probes is close, resulting in the so-called proximity effect. At this time, by adding a section of connector oligonucleotides that are complementary to the DNA attached to the antibody, the DNA on the PLA probe will be complementary to this section of DNA through pairing and complementation. Then, under the action of ligase, the fragments of DNA on the PLA probe were ligated together to form a new DNA fragment. The new DNA fragment can be amplified and quantified by fluorescent PCR, thus corresponding to the quantification of the target protein (Söderberg et al., 2006). As described above, aggregation intermediates produced by intracellular oligomerization can induce cytotoxicity. In neurodegenerative diseases these oligomers could diffuse extracellular and be taken up by neighboring cells, inducing conformational transitions of native α-Syn and aggregation deposition in recipient cell (Danzer et al., 2012; Guo and Lee, 2014). The technique has been successfully used for detection of α-Syn oligomers in post-mortem brain tissue from PD patients, even in early-stage disease. Roberts et al. initially established α-synuclein-PLA and then demonstrated sensitive protein assays to recognize α-synuclein oligomers but not monomeric nor high molecular weight species (Roberts et al., 2015). Ruffman and colleagues applied this assay to gastrointestinal tissue (Ruffmann et al., 2018). Oligomerized-α-Syn was found in most tested sections in this study, but PLA couldn’t demonstrate a distinctive pattern or quantitative threshold to discriminate PD patients from healthy controls. Oligomerized α-Syn on synaptic terminals of the skin was reported by PLA in a recent case-control study, with a sensitivity of 82% and a specificity of 89% (Mazzetti et al., 2020). α-Syn PLA has the potential for the detection of α-synucleinopathies, however, further studies are needed in the future to verify its utility along with the role of oligomerized-α-Syn in disease progression.

### Proteinase K digestion of α-Syn

Resistance to PK digestion is defined as a misfolded α-Syn. Tissue sections and lysates were pre-treated with PK, which can digest and eliminate non-pathological α-Syn. These assays imitate the pathological conversion of the native α-Syn into a PK-resistant-associated conformer, which makes up for the shortcomings of traditional methods in distinguishing the pathological and physiological morphology of α-Syn. Different bands of tissue lysates from different α-synucleinopathies pre-treated with PK can be detected by immunoblotting, revealing a different strain of α-Syn with different PK resistance, which could be used for α-Syn strain subtyping. Furthermore, the paraffin-embedded tissue (PET) blot technique, originally established for pathological prion protein detection in fixed tissues, combines the advantages of the high sensitivity of immunoblotting and high anatomical resolution of IHC. However, although PK-PET blotting could retain the anatomical resolution of the test tissue, extended digestion can make it highly variable and reduce the integrity of the tissue; therefore, the neuroanatomical resolution to be analyzed is very poor.

### Enzyme-linked immunosorbent assays

Enzyme-linked immunosorbent assays (ELISAs) is also used in the pathological α-Syn detection, especially in various biological fluids from patients with PD. Several studies have used ELISA to explore α-Syn oligomers by the same epitope-blocking antibody to capture and detect them. Blocking antibodies are used so that only one single antibody molecule binds to each α-Syn molecule, avoiding monomeric signals. Several studies have found higher levels of α-Syn oligomers in plasma and CSF from PD patients than in controls, suggesting that α-Syn oligomers in biological fluids may be promising biomarkers for PD patients (El-Agnaf et al., 2006; Tokuda et al., 2010). However, measurement of total levels of α-Syn often masks differences in oligomer levels because multiple α-Syn conformations are detected (Tokuda et al., 2010). In addition, the α-Syn level in the saliva from PD patients was lower than that of healthy controls as measured by ELISA (Al-Nimer et al., 2014). However, the methods described so far indicate that there is a great deal of variability and overlap in ELISA signals between the controls and patients with PD. Therefore, such techniques as a biomarker need to be further explored.

### Protein-misfolding cyclic amplification

Protein-misfolding cyclic amplification (PMCA) was originally applied for the detection of pathologic prion proteins with high sensitivity and specificity (Saborio et al., 2001; Saá et al., 2006). This assay is built on earlier observations in which infectious prion protein aggregates, such as PrP^Sc^, induced the transformation of native cellular prion protein (PrP^C^) to an aberrant protease-resistant form with significant species and strain specificity (Raymond et al., 1997, 2000). Newly formed misfolded subunits extend and aggregate to form large fibrillary structures. Ultrasonication is used to decompose the misfolded aggregates into more “seeds,” which react again in a circular fashion. By the end of round one of PMCA, most PrP^C^ molecules are converted into their misfolded form, and then the product is treated with proteinase K (PK) and then PK-resistant fragments are monitored through Western blot detected by an anti-PrP antibody (Saborio et al., 2001; Atarashi et al., 2007). Due to the prion-like properties of α-Syn, PMCA can also be used in synucleinopathies. Many reports have pointed out that PMCA assay is an extremely sensitive and quantitative method for detecting α-Syn seeding (Saborio et al., 2001; Saá et al., 2006; Shahnawaz et al., 2020). Using α-Syn PMCA to a blinded analysis of a group of PD, DLB, MSA and control CSF samples, the sensitivity of this study was 88.5%, 100%, and 80%, respectively, and the specificity was 96.9% (Shahnawaz et al., 2017). It was also found that the unique biophysical properties of α-Syn seeds, such as PK resistance, were retained after PMCA detection. The PMCA assay has a strong upside, but still has a few drawbacks. One common drawback of PMCA is the need for serial cycles of sonication in a case-by-case manner for maximum amplification, which is difficult and expensive to maintain, making this technique highly unsuitable as a high-throughput diagnostic assay in hospitals or other medical centers.

### Real-time quaking-induced conversion

**FIGURE 2:**
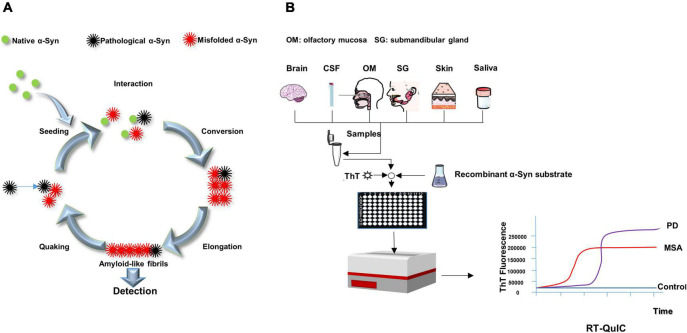
Schematic presentation of RT-QuIC mechanism and process. **(A)** The pathological α-Syn seeds from patients (black) trigger conformational changes of native α-Syn (substrate, green) to form new misfolded α-Syn (red). The misfolded α-Syn proteins aggregate into oligomers, further elongating into fibrils. After the fibrils are detected, the quaking breaks the fibrils into shorter oligomers (seeds), which combine with other native α-Syn to promote the transformation again. **(B)** Samples are mixed with reaction buffer, including α-syn substance and thioflavin T (ThT, a fluorescent probe), loaded into a multi-well microplate and incubated in alternating cycles of shaking and rest in a fluorescence microplate reader. The device records real time ThT fluorescence emission. A sigmoidal growth in ThT fluorescence reveals synucleinopathies. The difference of lag period and maximum of ThT fluorescence could be used to distinguish MSA from PD.

Shortly after the invention of the PMCA, a simpler and more manageable technique, RT-QuIC, was developed. The RT-QuIC method can specifically bind thioflavin T (ThT) using transformation products rich in β-sheet secondary structures. Therefore, recombinant α-Syn undergoes conformational transformation upon contact with misfolded α-Syn (seed) and further extends to grow into amyloid fibrils, which can bind to ThT and thus, can be monitoring real-time by measuring the fluorescence of ThT as relative fluorescence units (RFU) over time (Figure 2A). RFU-related parameters such as lag phase can reflect α-Syn seeding activity. The lag phase is the time it takes for a reaction to reach a threshold that can be used to distinguish between negatives and positives. Protein aggregation rate (PAR) was defined as the inverse of lag phase (1/h) and could be used to compare seed activity at various dilutions (Cramm et al., 2016; Cheng et al., 2017). And some other RT-QuIC kinetic parameters, including maximum ThT fluorescence (Fmax), time to Fmax (T50), and area under the curve (AUC), could be also utilized to discriminate synucleinopathy cases from controls. For example, Rossi et al. showed that CSF α-Syn RT-QuIC associated with LB- pathology had higher Fmax and AUC, which could distinguish them from non-LB pathology (Rossi et al., 2020). Some RT-QuIC studies have also demonstrated the utility of using PAR parameters in distinguishing synucleinopathies from controls (De Luca et al., 2019; Manne et al., 2019, 2020a,b; Bargar et al., 2021b). In addition, PAR was significantly higher in DLB than in PD over several orders of magnitudes of brain homogenate dilutions, reflecting higher levels of α-Syn aggregates in the brain of DLB patients than in PD patients (Bargar et al., 2021b).

Because this method is capable of detecting PrP^Sc^ as low as the fg grade, its high sensitivity qualifies this technique for achieving ante-mortem or non-invasive clinical diagnosis of prion proteins by detecting PrP^Sc^ in various tissues and biological fluids. RT-QuIC is significantly better than other assays in terms of repeatability, practicability, rapidity and low cost. It is more convenient than other detection methods, such as the use of sonication in PMCA to break the fibrils into more “seeds” at regular intervals. In PMCA, the reaction system is incubated and sonicated in a water bath with a sonicator; the tubes positioned at the periphery of the sonicator show less efficacy of amplification compared to those positioned in the center. RT-QuIC, on the other hand, uses intermittent vibrations to generate more “seeds,” which seem to be easier to control. In addition, RT-QuIC was performed in tape-sealed microplates, which minimized the risk of aerosol contamination per well. The multi-well format can analyze up to 96 samples in the same experiment. In addition, no sequence homology is required between seeds and substrates in RT-QuIC. RT-QuIC readings are ThT fluorescence values (RFU), which increase with substrate conversion to amyloid, whereas PMCA readings are immunoblots or immunoassays (Saborio et al., 2001; Gonzalez-Montalban et al., 2011; Cheng et al., 2017). Given the many advantages of RT-QuIC, the assay is easy to practical use in the clinic, helping clinicians with the diagnosis and monitoring of treatment progress (Figure 2B).

Realtime quaking-induced conversion (RT-QuIC) was first developed and validated using brain tissue and CSF from PD and DLB patients. Fairfoul et al. (2016) first successfully applied α-Syn RT-QuIC to detect α-Syn aggregation in brain tissue and CSF from synucleinopathies. The results showed sensitivities of 92% and 95%, respectively, with an overall specificity of 100%. Subsequently, multiple teams have observed high specificity and sensitivity of α-Syn RT-QuIC in CSF and brain tissue (Groveman et al., 2018; Sano et al., 2018; Bongianni et al., 2019; Garrido et al., 2019; Kang et al., 2019; van Rumund et al., 2019; Han et al., 2020; Rossi et al., 2020; Bargar et al., 2021b; Brockmann et al., 2021; Iranzo et al., 2021; Perra et al., 2021; Poggiolini et al., 2021). Recent studies applied RT-QuIC to submandibular gland, biopsies of skin, and olfactory mucosa, which indicates a growing trend toward non-invasive testing (De Luca et al., 2019; Manne et al., 2020a,b; Wang et al., 2020; Bargar et al., 2021a; Kuzkina et al., 2021; Stefani et al., 2021; Luan et al., 2022). The results of protease resistance of RT-QuIC products showed that seeded fibrils had obvious disease-specific protease sensitivity in PD and MSA, indicating that RT-QuIC amplification could preserve the structural features of the initial seeds and help to differentiate between different strains (Manne et al., 2020b). Significant progress has been made in α-synucleinopathy diagnosis with the development of RT-QuIC (Bargar et al., 2021b). Hence, once optimized and integrated into clinical practice, RT-QuIC has the potential to be validated to refine diagnosis, design clinical trajectories, monitor disease progression, and recruit subjects eligible for targeted therapies.

## Tissues and biofluids investigated for detection of α-Syn seeding

**FIGURE 3:**
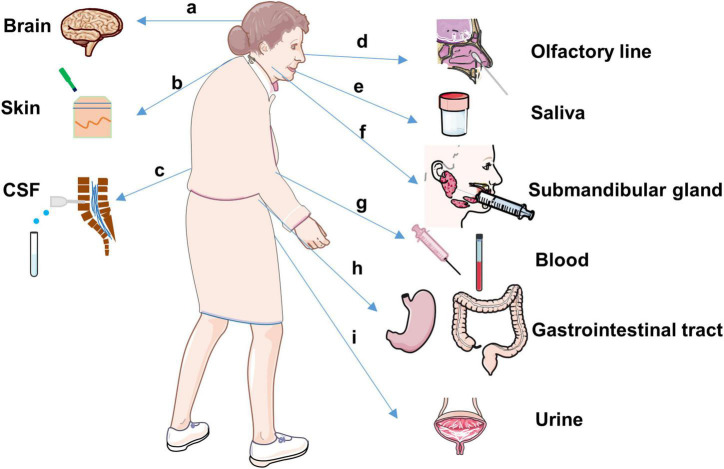
Tissues and biofluids in α-synucleinopathies for α-Synulein seeding activity detection.

Detection of α-Syn-seeding activities in biological fluids or peripheral tissues is of particular interest because of its potential use as a biomarker for α-synucleinopathies. Recently, seed amplification assays through the prion-like properties of misfolded α-Syn have shown an ultrasensitive in diverse tissues and biological fluids, such as the brain, CSF, gastrointestinal tract, skin, olfactory mucosa, salivary, etc. (Figure 3).

### Brain

During PD progression, Braak et al. first proposed that pathologic α-Syn may propagate between brain regions. According to Braak’s model, LB and LN formation begins early in the disease (even before motor symptoms appear), originating in the olfactory bulb and brainstem, particularly in the dorsal motor nucleus of the vagus nerve (Braak et al., 2003). At the same time as the disease progresses, LB and LN can be detected in other parts of the brain region, such as the midbrain and even the cerebral cortex, suggesting that the prion-like diffusion process of α-Syn appears to begin in the brain. Brain tissue is the strongest sample for detecting α-Syn seeding characteristics. Several reports have shown that α-Syn seeding activity is highly sensitive and specific in PD and DLB brains (Groveman et al., 2018; Bargar et al., 2021b). Of note, several studies have reported that distinct misfolded proteins co-exist in some neurodegenerative diseases; Specifically, the brains of some neuropathologically proven tauopathies, such as AD, progressive supranuclear palsy (PSP), and corticobasal degeneration (CBD), have been reported to contain LBs (Mori et al., 2002).

### Cerebrospinal fluid

Detection of CSF α-Syn aggregation is a promising biomarker for the diagnosis of α-synucleinopathies. Several studies have shown that oligomeric α-synuclein is toxic and plays an important role in the neuropathology of PD. CSF oligomeric α-Syn has been consistently found at a higher concentration in patients with PD compared with controls, with a comprehensive sensitivity of 71% and a specificity of 64%, but its low diagnostic accuracy limits its application in clinical diagnosis (Eusebi et al., 2017). Few studies have reported higher concentrations of phosphorylated α-Syn in CSF of patients with PD than in controls (Eusebi et al., 2017).

Seeding aggregation assays such as PMCA and RT-QuIC have been shown to detect and measure α-Syn pathogenic aggregation in CSF. In 2018, Groveman et al. (2018) performed RT-QuIC on CSF samples from patients with PD, DLB, and non-synucleinopathies, including patients with Alzheimer’s disease and healthy individuals. In DLB and PD patients, 93% of individuals had positive RT-QuIC responses, whereas non-synucleinopathy controls were not, yielding 100% specificity. Subsequently, in 2020, Rossi et al. used a relatively large cohort to study CSF-

α-Syn RT-QuIC as a means of the detection of Lewy Body (LB)-related disorders and control, achieving an overall sensitivity of 95.3% and a specificity of 98% (Rossi et al., 2020). In 2021, Brockmann et al. (2021) utilized CSF from a group of patients with clinically diagnosed sporadic PD/DLB and familial PD/DLB patients with different mutations such as GBA(PD/DLB) and LRRK2(PD). α-Syn RT-QuIC was positive in 87% of patients with *GBA* mutations, and 78% of patients with *LRRK2* mutations, which might provide a basis for RT-QuIC technique to help the diagnosis of familial parkinsonism. IRBD is a prodromal risk factor for α-synucleinopathies, which precedes typical PD symptoms such as motor symptoms and dementia. Rossi’s team achieved 100% sensitivity and 100% specificity by detecting seeding activity with α-Syn RT-QuIC in all 18 iRBD patients but not in all 11 control patients. The results from Rossi suggest that seeding aggregation assays may be useful for detecting precursor α-synucleinopathies. In addition to studies distinguishing synucleinopathies from non-synucleinopathies, Shahnawaz and colleagues applied PMCA to compare CSF from patients with PD and MSA.

### Gastrointestinal tract

Approximately 60% of patients with PD experience gastrointestinal symptoms, such as constipation, many years before the occurrence of the common movement disorder, thus making gastrointestinal tract tissues suitable for the early diagnosis of PD. In recent years, there have been many reports on the brain-gut axis. Results of an *in vivo* study from Ulusoy revealed a route-specific transmission of α-Syn from the rat brain to the stomach (Ulusoy et al., 2017). However, Dawson and others suspected that the gastrointestinal tract may be the origin of PD since α-Syn could spread from the gut to the brain *via* the vagus nerve (Kim et al., 2019). Recently, Challis and colleagues found that inoculating the duodenal wall of mice with preformed fibrils (PFFs) led to changes in the enteric nervous system and gastrointestinal deficits (Challis et al., 2020). Ultimately, inoculation of α-Syn PFFs resulted in the progression of α-Syn histopathology to the midbrain in aged mice. In recent years, α-Syn-seeding detection in biopsy specimens by PMCA and RT-QuIC has been performed, which revealed that these methods can detect α-Syn aggregates in routine gastrointestinal biopsies (Fenyi et al., 2019).

### Submandibular gland and salivary

α-Syn was found to be present in the submandibular glands in most PD cases in a post-mortem autopsy-based study (Beach et al., 2016). With the high amount of α-Syn deposition in autopsy studies, the submandibular gland has become a promising site for biopsies in PD. Manne et al. (2020a) were the first to apply RT-QuIC assay on peripherally submandibular gland tissues to detect α-Syn seeding in PD patients with high sensitivity and specificity (Manne et al., 2020a). Manne et al. also found that the RT-QuIC assay, on submandibular gland tissues, provides higher sensitivity (100%) and specificity (94%), superior to phosphorylated α-Syn IHC of the minor salivary glands. Although with the high sensitivity and specificity, the invasive nature of the sampling procedure may limit its clinical application. Submandibular gland biopsies could lead to serious rare adverse events, such as infection, hematoma, and facial nerve damage. Therefore, it is necessary to develop more minimally invasive and even non-invasive samples to help clinical diagnosis.

More recently, the results of saliva α-Syn RT-QuIC assay by Luan et al. (2022) showed that the sensitivity of α-Syn RT-QuIC assay for PD patients was 76.0%, the specificity was 94.4%, and the sensitivity for MSA patients was 61.1%. Although the sensitivity of saliva RT-QuIC detection is not very high, saliva is still very attractive as a non-invasive test sample. Therefore, salivary α-Syn seeding activity may be a promising biomarker for the diagnosis of PD and MSA. However, further studies are needed to verify the utility of salivary RT-QuIC for clinical diagnosis in the future.

### Skin

α-Syn deposition in skin biopsies of PD patients was first reported in 2005; since then, the skin was considered as a favorable site for PD. Early researches demonstrated the possibility of detection of phosphorylated α-Syn in skin tissues using IHC. However, subsequent positive rates of phosphorylated α-Syn aggregates in histopathological studies varied dramatically. Biopsies from the same site could show significantly different results (from 0 to 100%). Wang et al. demonstrated for the first time that skin α-Syn has a meaningfully higher aggregation activity in patients with PD and other synucleinopathies than in control patients such as tauopathies and non-neurodegenerative diseases (Wang et al., 2020). The panel also found that RT-QuIC sensitivity (93%) was higher than sonicated PMCA (82%) in differentiating autopsied skin samples from clinically diagnosed PD patients and controls. Manne et al. (2020b) collected scalp skin tissue from PD patients and controls, using only post-mortem samples, and found that seeding activity was significantly stronger in PD patients than in controls, with an overall sensitivity of 96% and specificity of 96%. The feasibility of using skin tissue for PD diagnosis was further demonstrated in another study, which reported high RT-QuIC sensitivity to skin punches from post-mortem (88.9%) and live patients (89.3%) (Mammana et al., 2021). He also proposed that the skin-based analyses provided comparable diagnostic sensitivity and specificity to CSF-based assays. Another study from Kuzkina showed α-Syn seeding activity by RT-QuIC was higher in patients with longer disease duration and more advanced disease stage, and was associated with rapid eye movement (REM) sleep behavior disorders, cognitive impairment, and constipation. This suggests that α-Syn RT-QuIC seeding activity in the skin may serve as a potential indicator of progression as it is correlated with disease stage and certain non-motor symptoms (Kuzkina et al., 2021).

### Olfactory mucosa

Olfactory dysfunction precedes the onset of motor or cognitive symptoms in PD and other α-synucleinopathies by several years. Therefore, it is crucial to determine whether the olfactory bulb is one of initially affected areas of misfolded α-Syn, which could help to understand how α-synucleinopathies evolve. Rey et al showed misfolded α-Syn species, injected into the olfactory bulb of wild-type mice, could rapidly transport throughout the olfactory system and interconnected brain regions (Rey et al., 2013). Widespread synucleinopathy was rapidly transferred to both direct and indirect brain regions of mice connected to the injection site over 6 months after injection of α-Syn preformed fibrils unilaterally into the olfactory bulb (Mezias et al., 2020). Thus, olfactory mucosa has received attention as a potential early biomarker for neurodegenerative disease.

Recent evidence has demonstrated that abnormal α-Syn can accumulate in the olfactory epithelium collected post-mortem from PD patients. Olfactory mucosa samples obtained by nasal brushing testing of patients with PD and MSA were analyzed by α-Syn RT-QuIC. The results showed that most patients with PD and MSA could efficiently induce α-Syn aggregation, but some control patients also found increased α-Syn aggregation activity, resulting in low specificity of detection by olfactory mucosa samples. Although the accuracy of olfactory mucosa in the diagnosis of PD is not very high, the combination of olfactory mucosa and CSF RT-QuIC analysis can significantly improve the overall diagnostic accuracy of these diseases, especially in the early stage (De Luca et al., 2019). Stefani et al. (2021) demonstrated that 44% of isolated RBD patients and 46% of PD patients were positive for α-Syn RT-QuIC seeding activity in olfactory mucosa, with an overall specificity of 90%. Interestingly, isolated RBD patients with positive α-Syn seeding activity were more likely to have olfactory dysfunction than those without seeding activity (79% vs. 23%). Another study by Perra et al. (2021) used α-Syn RT-QuIC to achieve the highest diagnostic accuracy to date in olfactory mucosa. The results showed an overall accuracy of 86.4%, a sensitivity of 81.4%, and a specificity of 92.1%.

Although the overall sensitivity was not particularly high in α-synucleinopathies, nasal brushing testing remains attractive as a simple, non-invasive sample that may be used as part of an early screening regimen to identify patients in the pre-clinical and prodromal phases. While α-Syn-seeding activity can be observed in the CSF, submandibular gland, and gastrointestinal tract, these methods are invasive, and both the feasibility and patient acceptance are limited when it involves large-scale screening for prodromal PD or repeated prospective assessment for disease progression. The advent of nasal brushing makes it easier for patients to cooperate. Nasal brushing testing is non-invasive, easy to perform, and can be repeated at all stages of the disease without any sequelae. Furthermore, because of the emergence and spread of COVID-19, nasal brushing testing has also been accepted worldwide. However, further studies are needed to enhance the sensitivity and offer a better understanding of the temporal dynamics of α-Syn seeding in the olfactory mucosa, as well as its spread to other brain areas during the progression from idiopathic RBD (iRBD) to overt α-synucleinopathy. More knowledge is required on the impact of disease progression, disease subgroups, and sampling techniques on the overall sensitivity.

### Blood

In a longitudinal study, increased phosphorylated α-Syn, but not total α-Syn, was detected in PD plasma samples compared with samples from healthy controls. The α-Syn oligomer in plasma samples obtained from PD patients significantly increased compared with that of controls, and ELISA revealed a specificity of 85.2%, a sensitivity of 52.9%, and a positive predictive value of 81.8% (El-Agnaf et al., 2006). Wang et al. demonstrated that patients with PD can increase α-Syn neurotoxicity by promoting phosphorylation and oligomerization of plasma extracellular α-Syn (Wang et al., 2016). Another study from Williams also showed that oligomeric α-Syn variants were preferentially present in PD brain tissue, CSF, and serum, and the sensitivity and specificity of serum to correctly identify α-Syn oligomers in patients with PD were 75% and 100%, respectively (Williams et al., 2016). Similarly, in addition to oligomerized α-Syn, the phosphorylated α-Syn in PD group was significantly higher than that in control group (Foulds et al., 2013). Misfolded prion can be identified by RT-QuIC in the blood and urine of people with Creutzfeldt-Jakob disease (CJD), reaching sensitivities and specificities approaching 100%. However, there is no report to verify the α-Syn RT-QuIC assay in the blood samples from α-synucleinopathies. Considering the existence of misfolded α-Syn in blood of α-synucleinopathies and the successful application of blood in clinical practice for CJD (Foulds et al., 2013; Williams et al., 2016), blood may be a practical sample for RT-QuIC assay and more future experimental studies should focus on less invasive sample sources.

### Urine

Recent studies suggest that 30–40% of persons with PD have urinary symptoms, suggesting that urine may be used as a non-invasive and reproducible sample to detect pathological α-Syn for clinical diagnosis. Distinct oligomeric formations of α-Syn oligomers were detected in the urine of PD diagnostics by ELISA. Nam et al. found increased α-Syn oligomer in the urine of PD patients (Nam et al., 2020); this measure had weak correlations with the index of PD progression. There is no report on α-Syn RT-QuIC or PMCA in the urine of persons with α-synucleinopathies.

## Application of α-Syn seeding in the diagnosis of synucleinopathies

### Distinguishing synucleinopathies from non-synucleinopathies and healthy controls

In recent years, several research teams have successfully identified synucleinopathies from control patients via seeding aggregation assays. A blinded study of CSF samples by PMCA technology correctly identified PD patients with an overall sensitivity of 88.5% and a specificity of 96.9% (Shahnawaz et al., 2017). Fairfoul et al. (2016) found that seeding activity in PD patients was significantly increased by RT-QuIC, which can discriminate PD from controls and AD (sensitivity 95%, specificity 100%). Wang et al. (2020) conducted a research evaluating skin from autopsy and biopsy specimens. They concluded that RT-QuIC and PMCA analysis of skin α-Syn seeding activity in patients with parkinsonism can distinguish synucleinopathies (PD, MSA, and LBD) from tauopathies (PSP and CBD) and the RT-QuIC assay is more sensitive than PMCA. In conclusion, PMCA and RT-QuIC have high sensitivity and specificity in distinguishing synucleinopathies from non-synucleinopathies and healthy controls.

### Differential diagnosis within different synucleinopathies

The neuroanatomical distribution, morphological and biochemical characteristics of α-Syn aggregates differ between MSA and PD, and the reason for this variability seems to be caused by different strains of α-Syn (Miki et al., 2019). For example, α-Syn-induced aggregates obtained from glial cytoplasmic inclusions in MSA have different properties than those seeded from LB-derived α-Syn (Peng et al., 2018). An earlier study by Groveman et al. showed that propagation of PD and DLB seeds resulted in fibrillation products with different ThT amplitudes (Groveman et al., 2018). In 2019, De Luca further demonstrated that the biochemical and morphological properties of α-Syn fibrils generated by RT-QuIC differ significantly between PD and MSA and are useful for disease discrimination (De Luca et al., 2019). Consistent with previously published findings, Shahnawaz et al. concluded that the maximum fluorescence and aggregation kinetics of PD and MSA were consistently different, with samples from MSA patients aggregating faster but reaching a lower fluorescence plateau than those from patients with PD, providing better discrimination between α-Syn strains in PD and MSA (Shahnawaz et al., 2020). The report also showed structural differences between PD-and MSA-amplified aggregates using techniques such as circular dichroism, FTIR, and cryo-electron tomography. Singer et al. also support the ability of PMCA to distinguish between MSA and PD/DLB. He found that levels of neuromerifilament light (NFL) (a marker of neuronal degeneration, but not disease-specific) were significantly higher in early MSA compared with early PD or DLB. In addition, consistent with Shahnawaz’s study, the maximum ThT fluorescence of PD and DLB samples was larger than that of MSA samples, but the MSA samples started polymerization and formation of reaction platforms earlier. Another major clinical variation is the presence of two different MSA phenotypes, Parkinsonian type (MSA-P) and cerebellar type (MSA-C). Bargar et al. (2021a) support the hypothesis that MSA-P and MSA-C may be caused by distinct α-Syn strains. They found that effective α-Syn RT-QuIC seeding activity was observed in olfactory mucosa of PD and MSA-P patients, but not in those of MSA-C patients.

### Application of α-Syn seeding in pre-clinical α-synucleinopathies

The identification of the early or prodromal phase of α-synucleinopathies has been a major concern for disease modification and neuroprotective therapeutic approaches. α-Syn-seeding activity is detected in clinical syndromes that can precede PD and cognitive disorders, including iRBD and pure autonomic failure (PAF). Manne et al. (2020a) showed that RT-QuIC can detect misfolded α-Syn in submandibular gland tissues from incidental Lewy body disease (ILBD) patients that was not detected by IHC, suggesting that the RT-QuIC assay has the potential to identify prodromal PD in submandibular gland tissues. A large cohort study by Stefani et al. found that 44.4% of patients with iRBD, 46.3% of patients with PD, and 10.2% of controls were positive for α -Syn RT-QuIC seeding in olfactory mucosa, with an overall specificity of 89.8%, a sensitivity of 45.2%, and an accuracy of 61.3% (Stefani et al., 2021). Mild cognitive impairment (MCI) is also common in early-stage of DLB. The overall sensitivity, specificity and accuracy of CSF-α-Syn RT-QuIC were 95.1%, 96.6% and 96%, respectively, in distinguishing MCI samples with Lewy bodies from controls without cognitive impairment, indicating that RT-QuIC may be a promising assay in the diagnosis of prodromal α-synucleinopathies (Rossi et al., 2021). Another study using a blinded patient cohort to detect simultaneously CSFs and OMs in patients showed a sensitivity of 81.4% and a specificity of 92.1% for identifying probable or prodromal DLB cases (Perra et al., 2021). Iranzo et al. (2021) showed that pathological α-Syn is detectable in CSF years before the clinical onset of PD or DLB during long-term follow-up, suggesting that the α-Syn detected in CSF RT-QuIC is a precursor marker. However, Rossi et al. (2020) found positive test responses in 4 from the healthy controls and 16 iRBD patients did not develop any clinical manifestations indicative of synucleinopathy at the end of the study. Some participants with a sub-clinical PD or DLB who did not evolve into α-synucleinopathy during the observational period were possible, and further investigation is needed. Based on the discussion above, α-Syn RT-QuIC may provide a valuable marker for recognizing patients in an early stage of α-synucleinopathies, and might help recruit patients for clinical disease modification trials of interventions targeting α-Syn seeding.

## Therapies targeting α-Syn seeding

**FIGURE 4:**
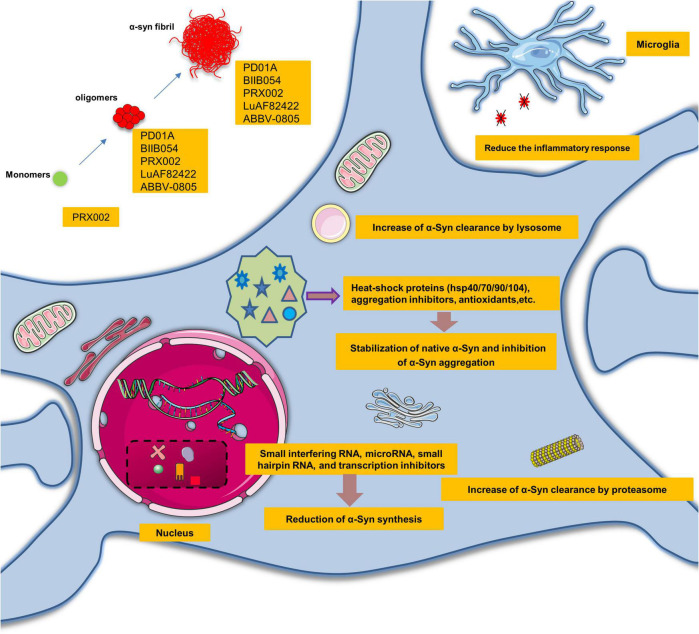
Promising therapeutic perspectives and clinical trials targeting α-synuclein seeding. α-Syn-directed therapeutics might require alleviating the neurotoxic gain of α-Syn seeding in several ways: reduction of α-Syn synthesis, stabilization of native α-Syn, inhibition of α-Syn aggregation, and increase of α-Syn clearance.

In recent years, several studies have shown that the pathogenicity of α-Syn is related to the increased synthesis of α-Syn, the aggregation of misfolded α-Syn or the disturbance of α-Syn clearance. Therefore, α-Syn-directed therapy may be required to mitigate α-Syn neurotoxicity in several ways: reducing α-Syn synthesis, stabilizing native α-Syn, inhibiting α-Syn aggregation and increasing α-Syn clearance (Figure 4).

Small interfering RNA, microRNA, small hairpin RNA, transcription inhibitors, and salbutamol can reduce the synthesis of α-Syn (Lewis et al., 2008; Mittal et al., 2017). Some substances also reduce the aggregation of α-Syn via heat-shock proteins (hsp40/70/90/104), aggregation inhibitors, and antioxidants. For instance, hsp90 chaperone has been reported to neutralize α-Syn complexes in an ATP-dependent manner (Danzer et al., 2011; Daturpalli et al., 2013). Perhaps the most promising strategy is the use of small molecules that would have the potential to alter the conformation of proto-fibrillar forms of α-Syn and render them non-pathogenic. The green tea derivative EGCG is an example of this natural product (Bieschke et al., 2010).

Furthermore, oligomerization can also be restrained by certain small molecules such as Anle138b (Wagner et al., 2013; Levin et al., 2014). Another therapeutic approach is to promote the activity of the ubiquitin-proteasome system and autophagy lysosomal pathway to increase the clearance of α-Syn. For example, several studies have demonstrated the neuroprotective effects of stimulation of autophagy through overexpression of transcription factors (Malagelada et al., 2010; Xilouri et al., 2013). Rapamycin or trehalose may be potential approaches to increase autophagy function and increase clearance of protein aggregates (Bové et al., 2011). There is also preliminary evidence that poly(DL-lactide-co-glycolide) acidic nanoparticles could restore impaired lysosomal function and promote α-Syn degradation in a range of PD models (Bourdenx et al., 2016). TTR is also a prion-like protein, associated with numerous diseases, including familial amyloid cardiomyopathy and familial amyloid polyneuropathy. TTR is a highly stable tetramer structural protein. Under pathological conditions (such as stress and inflammation), the tetramer can be decomposed and degraded into monomer, and then the monomer can be transformed and abnormally aggregated to form amyloid fibers, causing cytotoxicity. In recent years, FDA has approved some drugs (Tafamidis, Acoramidis, and NI-006) for the treatment of TTR-mediated amyloidosis, which can stabilize its tetramer conformation, suppress protein misfolding, and reduce cardiovascular mortality and the frequency of cardiovascular-related hospital stays in clinical trials. The successful development of TTR tetramer stabilization drugs provides a good reference for the development of misfolded α-Syn drugs.

The discovery of lymphatic vessels in the brain focused attention on the role of the immune system in the brain. Immunotherapies, including active and passive immunization, have a significant neuroprotective effect (Valera and Masliah, 2013). New immunotherapeutic peptides PD01A and PD03A were developed by AFFiRiS (Wien, Austria), using an active immunization approach. The antigenic peptide mimics an epitope of human native α-Syn. Data from *post hoc* analyses indicate that PD01-induced antibodies preferentially bind to both oligomeric and fibrillar α-Syn compared with monomers, and there was a reducing trend of oligomeric α-Syn levels in plasma as well as CSF upon treatment with PD01A at week 26. The results showed PD01A presented with a clear immune response, while PD03A made no difference compared to the placebo (McFarthing and Simuni, 2019).

UB-312 is another vaccine that targets a 12-amino acid sequence of C-terminal α-Syn (Nimmo et al., 2020). Antibodies produced with UB-312 were able to bind α-Syn oligomers as well as fibrils in autopsy brain tissue from patients with α-synucleinopathies. A Phase I clinical trial of the vaccine in patients with PD is currently underway. The experiment consisted of two parts: part A to assess the optimal dose; part B to evaluate the safety and tolerability of the drug. The expected completion date is June 2022.

Several groups have attempted to develop passive immunization for misfolded α-Syn. PRX002, developed by Prothena (Dublin, Ireland), was the first α-Syn-based treatment entering clinical trials in 2015. PRX002 is a humanized IgG1 monoclonal antibody, which has a higher affinity (>400-fold) for the aggregated form compared with that of monomers. In pre-clinical studies, it reduced α-Syn pathology and protected against cognitive and motor dysfunction in several animal models. In phase I studies (NCT02095171 and NCT02157714), a single ascending-dose PRX002 was determined to be well tolerable and safe. PASADENA is Roche’s (Basel, Switzerland) phase II clinical study (NCT03100149) evaluating PRX002 in patients with early PD (Pagano et al., 2021). In the first part of the study (phase IIa), in 2020, Roche announced that PASADENA failed to meet its main goal of delaying the progression of motor and non-motor symptoms measured by the Movement Disorder Society—Unified Parkinson’s Disease Rating Scale (MDS-UPDRS) total score. However, this antibody showed signs of efficacy on secondary and exploratory measures and included a reduction in disease progression. PRX002 reduced the decline of motor function by 35% when compared to placebo after 1 year of treatment, measured by the MDS-UPDRS part III. Patients treated with PRX002 also demonstrated a significant delay in time for clinical meaningful worsening of motor progression when compared with placebo over 1 year. The phase IIb study (PADOVA) of PRX002 has been launched following “positive signals of efficacy” in phase II.

BIIB054 (Cinpanemab) is another N-terminal α-Syn antibody developed by Neurimmune (Schlieren, Switzerland) and has been assessed in clinical trials. BIIB054 is highly selective for the aggregated form of α-Syn compared to the α-Syn monomer. BIIB054 was well tolerated in a completed phase I trial (Brys et al., 2019). A phase II study, SPARK (NCT03318523), has been conducted in 311 patients with early-stage PD. SPARK did not meet its primary and secondary outcome measures in the first year, resulting in the discontinuation of BIIB054 development and the termination of the SPARK study.

Other possible passive immunization therapies are being tested in phase I clinical trials (Table 1).

**TABLE 1:** α-Synuclein-based passive immunotherapies involved in clinical trials for Parkinson’s disease.

Antibody	Epitope	Binding	Phase	Disease stage	Status	Trial identifier	Outcomes
PRX002	aa115-126	Monomers + + Oligomers + + + Fibrils +	I-II	Hoehn and Yahr Stage I or II	In progress	NCT02095171(Ia) NCT02157714(Ib) NCT03100149(II)	Fail to slow symptom worsening Show other signs of effectiveness
BIIB-54	aa1-10	Monomers -/ + Oligomers + + + Fibrils + + +	I-II	≤ 2.5 on the Modified Hoehn and Yahr Scale	Terminated	NCT02459886(I) NCT03716570(I) NCT03318523(II)	Fail to meet primary outcome measuress Fail to meet secondary outcome measures
MEDI1341	aa102-130	Monomers + Oligomers + + + Fibrils + +	I	Healthy subjects	Completed	NCT03272165	Well tolerated
LuAF82422	aa112-117	Monomers 0 Oligomers + + Fibrils + + +	I	≤ 3 on the Modified Hoehn and Yahr Scale	Completed	NCT03611569	Well tolerated
ABBV-0805	aa121-127	Monomers + Oligomers + + Fibrils + +	I	≤ 2 on the Modified Hoehn and Yahr Scale	Withdrawn	NCT04127695	

## Conclusion and future perspectives

In this review, we discussed the α-Syn structure and thoroughly traced the mechanisms of pathological α-Syn seeding. Although α-Syn lacks the infectivity of prions, distinct α-Syn strains indeed incur an endogenous permissive template to enable transformation, in a manner similar to that of prion. The ongoing evaluation of α-Syn RT-QuIC as a potential biomarker in clinically accessible biological specimens (including CSF, skin, olfactory mucosa, salivary glands, and gastrointestinal tract) of α-synucleinopathies has an important role in understanding the disease pathogenesis, allowing for early and differential diagnosis. To date, the correlation between α-Syn RT-QuIC results and clinical data about disease progression and prognosis has been weak or insignificant. The high accuracy of the results of the amplification assays, coupled with the successful application in the diagnosis of CJD in clinical practice, indicates that the ultimate clinical application as a diagnostic test for PD is promising. In the future, disease-modifying clinical trials, accurate and early clinical diagnosis will be cumulatively necessary for early disease intervention. With a detailed understanding of seeding amplification assays, the detection of clinical specimens is increasingly inclined to non-invasive methods. The use of non-invasive samples such as peripheral biological fluids (urine, blood, tears) for seeding analysis is a potentially attractive option and a focus for future research. However, these methods need to be established and standardized to facilitate future clinical applications. The hypothesis that the clinicopathological heterogeneity of synucleinopathies is associated with distinctive α-Syn strains is supported by accumulatively experimental evidence from seeding assays and structural biology. As different strains of α-Syn are used as biomarkers, the possibility of accurate diagnosis is increasing.

Although RT-QuIC detection has many advantages, more studies are needed to improve its sensitivity in the future, such as further lowering the limit of detection and decreasing assay time for the accurate and ultrasensitive detection of α-Syn seeds in patients’ samples. The latest technique, real-time fast amyloid seeding and translocation (RT-FAST) was reported for detecting α-Syn seeding activity by a nanopipette for both seeding and sensing procedure. RT-FAST does remarkably shorten the time of seeding experiment, but also can provide quantitative information on the initial concentration of α-Syn seeds compared to RT-QuIC. More studies are needed to test the program on patients’ samples and the utility of the procedure for clinical diagnosis. To conclude, amplification techniques by α-Syn seeding could facilitate pre-symptomatic diagnosis, clinical diagnosis, and differential diagnosis, along with offering the possibility of measuring the effects of therapeutics in reducing α-Syn seed load in the future.

## Author contributions

YX and JY: conceptualization and writing—review and editing. JL, HL, HZ, SD, and TZ: writing—original draft. YY, YL, XZ, and YW: preparation of figures, table, and editing. All authors contributed to the article approved the submitted version.
